# *Decorin *and *TGF-β*_*1 *_polymorphisms and development of COPD in a general population

**DOI:** 10.1186/1465-9921-7-89

**Published:** 2006-06-16

**Authors:** Cleo C van Diemen, Dirkje S Postma, Judith M Vonk, Marcel Bruinenberg, Ilja M Nolte, H Marike Boezen

**Affiliations:** 1 Department of Epidemiology, University Medical Center Groningen, University of Groningen, Groningen, The Netherlands; 2 Department of Pulmonology, University Medical Center Groningen, University of Groningen, Groningen, The Netherlands; 3 Department of Medical Biology, University Medical Center Groningen, University of Groningen, Groningen, The Netherlands

## Abstract

**Background:**

Decorin, an extracellular matrix (ECM) proteoglycan, and *TGF-β*_*1 *_are both involved in lung ECM turnover. Decorin and *TGF-β*_*1 *_expression are decreased respectively increased in COPD lung tissue. Interestingly, they act as each other's feedback regulator. We investigated whether single nucleotide polymorphisms (SNPs) in *decorin *and *TGF-β*_*1 *_underlie accelerated decline in FEV_*1 *_and development of COPD in the general population.

**Methods:**

We genotyped 1390 subjects from the Vlagtwedde/Vlaardingen cohort. Lung function was measured every 3 years for a period of 25 years. We tested whether five SNPs in *decorin *(3'UTR and four intron SNPs) and three SNPs in *TGF-β*_*1 *_(3'UTR rs6957, C-509T rs1800469 and Leu10Pro rs1982073), and their haplotypes, were associated with COPD (last survey GOLD stage = II). Linear mixed effects models were used to analyze genotype associations with FEV_1 _decline.

**Results:**

We found a significantly higher prevalence of carriers of the minor allele of the *TGF-β*_*1 *_rs6957 SNP (p = 0.001) in subjects with COPD. Additionally, we found a significantly lower prevalence of the haplotype with the major allele of rs6957 and minor alleles for rs1800469 and rs1982073 SNPs in *TGF-β*_*1 *_in subjects with COPD (p = 0.030), indicating that this association is due to the rs6957 SNP. *TGF-β*_*1 *_SNPs were not associated with FEV_1 _decline. SNPs in *decorin*, and haplotypes constructed of both *TGF-β*_*1 *_and *decorin *SNPs were not associated with development of COPD or with FEV_1 _decline.

**Conclusion:**

Our study shows for the first time that SNPs in *decorin *on its own or in interaction with SNPs in *TGF-β*_*1 *_do not underlie the disturbed balance in expression between these genes in COPD. *TGF-β*_*1 *_SNPs are associated with COPD, yet not with accelerated FEV_1 _decline in the general population.

## Background

Chronic obstructive pulmonary disease (COPD) is characterized by irreversible airway obstruction and persistent airway inflammation. Transforming growth factor-β_1 _(TGF-β_1_) is one of the important cytokines involved in this inflammatory process, which has been associated with cell proliferation and differentiation. It is furthermore involved in repair of the extracellular matrix (ECM) after inflammation and tissue injury amongst others by promoting synthesis of elastin and collagen. Studies have shown that *TGF-β*_*1 *_expression is increased in the airways of COPD patients [1,2] In contrast, a recent article from Pons *et al *showed that alveolar macrophages from COPD patients release less *TGF-β*_*1 *_in response to lipopolysaccharide than smokers with normal lung function and non-smokers[3] This may lead to a reduced anti-inflammatory and anti-elastolytic response in COPD patients, subsequently contributing to progressive ECM destruction.

Decorin is a component of the ECM that regulates collagen fibrillogenesis. [4-6] In addition, it can interact with a wide variety of growth factors, cytokines and adhesion molecules through its extensive binding area, thereby not only playing a role in ECM assembly but also in control of cell proliferation and tissue morphogenesis.[7]*TGF-β*_*1 *_has been shown to downregulate synthesis of decorin in fibroblasts and decorin can in turn inhibit *TGF-β*_*1*_.[8] Decorin may thus act as a negative feedback regulator of *TGF-β*_*1 *_mediated repair responses. Conversely, *TGF-β*_*1 *_can downregulate expression of decorin in fibroblasts from emphysema patients.[9] We have shown previously that decorin expression is diminished in the peribronchiolar area of lung tissue from patients with severe emphysema, while *TGF-β*_*1 *_production from fibroblasts of these patients is increased.[10] Noordhoek *et al *showed that *TGF-β*_*1 *_and basic fibroblast growth factor give a stronger reduction of decorin production in the culture supernatant of fibroblasts from patients with severe emphysema than from patients with mild emphysema. [9] It thus appears that the regulation of decorin production is disturbed in lung tissue from patients with severe emphysema. This will lead to diminished binding and neutralization of *TGF-β*_*1 *_by decorin followed by higher *TGF-β*_*1 *_concentrations and activity with lower decorin production as a result.

We hypothesized that the reciprocal regulation of the *TGF-β*_*1 *_and *decorin *genes is disturbed in COPD due to a genetic mutation in one or both of these genes. We have tested this hypothesis by investigating three single nucleotide polymorphisms (SNPs) in *TGF-β*_*1 *_and five SNPs in *decorin *on the development of COPD and on lung function decline in a large cohort derived from the general population (the Vlagtwedde/Vlaardingen cohort).

## Methods

### Subjects

We used data from 2467 subjects of the Vlagtwedde/Vlaardingen cohort participating in the last survey in 1989/1990. This general population-based cohort of Caucasians of Dutch descent started in 1965. Surveys, during which pulmonary function measurements were performed, were held every three years. The selection of the cohort has been described previously. [11-13] Surveys were performed every 3 years during which information was collected on respiratory symptoms, smoking status, age and gender by the Dutch version of the British Medical Council standardized questionnaire. A blood sample was taken and spirometry was performed. Details on pulmonary function measurements are provided in the additional file 1. The methodology for standardization and equipment used for lung function measurements was the same throughout the study. In 1989/1990 neutrophil depot of centrifuged blood was collected and stored at -20°C. In 2003/2004 DNA was extracted from these samples with the QiaAmp^® ^DNA Blood Mini Kit and checked for purity and concentration with the NanoDrop^® ^ND-1000 UV-Vis Spectrophotometer. The study protocol was approved by the local university hospital's medical ethics committee and participants gave written informed consent.

### Genotyping

We genotyped DNA of those subjects with more than 1500 ng isolated DNA available (N = 1390). Three SNPs, previously associated with COPD or level of lung function were genotyped in *TGF-β*_*1*_: rs6957 in the 3'UTR, rs1800469 in the promoter region (C-509T) and a coding SNP rs1982073 (Leu10Pro, G/T). [14-16] Coding SNPs in *decorin *have been identified in the NCBI and Celera databases, but are only prevalent in African populations (frequency 0.05–0.12) but not in Caucasian populations (frequency 0.00). According to the HapMap database there are two large LD blocks in the decorin gene, and a region including the 3'UTR that forms no LD block. [17]. There are 4 haplotype tagging SNPs located in introns, resulting in 3 major haplotypes, which cover the information of the gene. Therefore, we genotyped one SNP in the 3'UTR (rs1803343), and the 4 haplotype-tagging SNPs: rs11106030, rs741212, rs566806, rs516115 and rs3138241. The genotyping protocol is described in the additional file 1; the characteristics of the genotyped SNPs in additional file 2. To determine whether the SNPs were in Hardy Weinberg equilibrium and whether they were in linkage disequilibrium, tests were performed with the statistical package R (version 1.9.1).

### Statistics

We identified subjects with COPD using the GOLD criteria (GOLD stage II or higher, i.e. FEV_1_/VC< 70% and FEV_1_<80% predicted) at the last survey[18] Characteristics of subjects with and without COPD at the last survey are presented in table 1. Differences in allele frequencies and haplotype frequencies between subjects with and without COPD were tested using Chi-square tests. We used ANOVA and linear regression models to study the effect of SNPs on first and last available FEV_1 _and FEV_1_/VC (adjusted for gender, age, pack-years, and height in regression models).

**Table 1:** Characteristics of genotyped subjects in the 1989/1990 survey

	No COPD (N = 1156)	COPD (N = 188)
Males, n (%)	554 (47.9)	137 (72.9)
Age in years, median (IQR)	50 (35–79)	59 (35–76)
Pack-years of smoking, median (IQR)	7.5 (0–21.6)	25.5 (6.6–35.7)
FEV_1_%pred, median (IQR)	95.8 (87.9–104.5)	71.1 (61.1–77.1)
FEV_1_/VC, median (IQR)	76.6 (62.1–80.5)	60.0 (54.5–65.7)

Abbreviations: FEV_1_, forced expiratory volume in 1 second; VC, vital capacity

Linear Mixed Effect (LME) models were used to investigate the effect of SNPs in *TGF-β*_*1 *_and *decorin *on annual FEV_1 _decline in the general population, like published previously.[19,20] Time was defined as time in years relative to the first FEV_1_, starting from the age of 30.[21] Variables included in the model were age at entry, gender, pack-years, the first FEV_1 _after age 30, and their interaction with time. Since including the level of the first FEV_1 _after age 30 and its interaction with time could introduce bias due to regression to the mean, these variables were also included in the model as random effect variables. The results of these analyses showed no change in estimates of the variables in the model or a better fit of the model, which indicates that there was no bias due to regression-to-the-mean. Therefore, the results are presented without these random effects. To test whether SNPs were associated with FEV_1 _decline within subjects with COPD, we performed LME analyses on these subjects only. Since Celedón *et al *found stronger linkage results of *TGF-β*_*1 *_SNPs and lung function in smokers only, we additionally performed LME models stratified to smoking status. [14] We also included interaction terms of *TGF-β*_*1 *_SNPs and *decorin *SNPs to test for genetic interaction of these SNPs.

Instead of performing pre- or post-hoc power analysis and correction for multiple testing, we performed permutation tests to assess whether our results might have been found due to chance. Genotypes were randomly shuffled among individuals to produce 3000 datasets. The LME models were rerun on each of these datasets to generate a distribution of the beta estimates for additional FEV_1 _decline of the homozygous minor allele genotype compared to FEV_1 _decline of the homozygous wild type allele genotype under the null hypothesis, being no association of the SNPs under study and FEV_1 _decline. If the observed beta estimate from the true data is found in the lower 5% percentile of the empiric cumulative distribution (p < 0.05), one can assume that the observed beta estimate is not found due to chance.

We also estimated *TGF-β*_*1 *_haplotype frequencies in the whole population and in subjects with a COPD phenotype. Estimated haplotype frequencies for *TGF-β*_*1 *_higher than 1% in the general population were used to construct phased multi-locus genotypes of *TGF-β*_*1*_. For *decorin*, we constructed the phased multi-locus genotypes as known from the HapMap database. With Chi-square tests we determined for each haplotype whether there was a difference in prevalence of carriers between subjects with and without COPD. Also, the excess decline in FEV_1 _in the whole population was determined for each phased multi-locus genotype in the LME.

Statistical analyses were performed using SPSS (version 12.0.1 for Windows), the statistical package R (version 1.9.1) and Arlequin [22].

## Results

Allelic frequencies for the minor alleles of the *TGF-β*_*1 *_and *decorin *SNPs in this population were comparable to those reported in the Celera and/or in the NCBI dbSNP database: *TGF-β1 *rs6957 0.18, rs1800469 0.28, rs1982073 0.38, *decorin *rs1803343 0.02, rs11106030 0.06, rs741212 0.12, rs566806 0.26, rs516115 0.22 and rs3138241 0.06. All SNPs were in Hardy Weinberg equilibrium. The *TGF-β*_*1 *_rs1800469 SNP was in significant LD with rs1982073 and rs6957. Rs6957 was in almost significant LD with rs1982072 (p = 0.06). The *decorin *SNPs were in significant LD. Graphs of the LD patterns with D', r and P-values in both genes are presented in the additional file 3.

### Prevalence of SNPs and haplotypes in *TGF-β*_*1 *_and *decorin *in COPD and control subjects

The distribution of the *TGF-β1 *rs6957 genotypes was significantly different between subjects with and without COPD (p = 0.001, table 2). The other *TGF-β*_*1 *_SNPs were not associated with COPD. We also found no association of SNPs in *decorin *with the prevalence of COPD.

**Table 2:** Prevalence of genotypes according to COPD phenotype (GOLD stage II or higher; FEV_1_/VC<70%, FEV_1 _<80% predicted).

**SNP**		**No COPD N (%)**	**COPD N (%)**	**P value df = 2**	**SNP**		**No COPD N (%)**	**COPD N (%)**	**P value df = 2**
TGF-β_1_	GG	584 (52)	106 (58)	0.541	Decorin	AA	878 (76)	131 (76)	0.913
rs1800469	GA	474 (40)	67 (36)		rs741212	AG	242 (22)	43 (22)	
	AA	87 (8)	10 (6)			GG	15 (2)	4 (2)	
									
TGF-β_1_	AA	382 (36)	75 (44)	0.297	Decorin	AA	614 (55)	102 (55)	0.949
rs1982073	AG	533 (49)	72 (42)		rs516115	AG	431 (38)	65 (38)	
	GG	156 (15)	23 (14)			GG	79 (7)	15 (7)	
									
TGF-β_1_	GG	771 (69)	103 (56)	**0.001**	Decorin	GG	863 (88)	136 (89)	0.733
rs6957	GA	327 (29)	71 (39)		rs3138241	GA	114 (12)	10 (11)	
	AA	30 (2)	10 (5)			AA	3 (0)	1 (1)	
									
Decorin	CC	996 (87)	170 (91)	0.217	Decorin	AA	1079 (94)	173 (93)	0.507
rs11106030	CA	142 (12)	8 (8)		rs1803343	AG	69 (6)	13 (7)	
	AA	4 (1)	1 (1)			GG	0 (0)	0 (0)	

Abbreviations: COPD, Chronic Obstructive Pulmonary Disease; FEV_1_, forced expiratory volume in 1 second; VC, vital capacity; *TGF-β*_*1*_, transforming growth factor-β_1_; df, degrees of freedom

We used estimated haplotype frequencies higher than 0.01 to construct phased multi-locus genotypes for *TGF-β*_*1*_. The haplotype consisting of the minor allele for *TGF-β*_*1 *_rs6957 and the wild type alleles for *TGF-β*_*1 *_rs1800469 and rs1982073 was more prevalent in subjects with COPD (p = 0.014). Because the prevalence of carriers of other haplotypes containing the minor allele at *TGF-β*_*1 *_rs6957 was also increased in subjects with COPD, this finding only reflects the individual association of the *TGF-β*_*1 *_rs6957 SNP with COPD. Carriers of at least one haplotype with the minor alleles for *TGF-β*_*1 *_rs1800469 and rs1982073 and the wild-type allele for rs6957 were less prevalent in COPD (p = 0.030). We found no significant associations of phased multi-locus genotypes in *decorin *with the prevalence of COPD (table 3). We also did not find associations of haplotypes containing SNPs of both *TGF-β*_*1 *_and *decorin *with COPD (data not shown).

**Table 3:** Prevalence of *TGF-β*_*1 *_and *decorin *haplotypes in subjects with and without COPD (GOLD stage II or higher; FEV_1_/VC<70%, FEV_1 _<80% predicted).

	Carrier of Haplotype*						
**TGF-β_1_**	**rs1800469**	**rs1982073**	**rs6957**		**No COPD N (%)**	**COPD N (%)**	**P value ^#^**

	0	0	0		239 (23)	34 (22)	0.686
	0	1	0		106 (11)	11 (7.6)	0.264
	0	1	1		27 (3)	6 (4)	0.417
	1	1	0		288 (29)	31 (20)	**0.030**
	0	0	1		95 (9)	25 (16)	**0.014**
	1	1	1		160 (16)	34 (22)	0.086

**Decorin**	**rs3138241**	**rs516115**	**rs714212**	**rs11106030**	**No COPD N (%)**	**COPD N (%)**	**P value**

	0	0	0	0	1009 (93)	175 (92)	0.515
	0	1	1	0	234 (22)	47 (27)	0.715
	1	1	0	1	133 (12)	15 (9)	0.950

Abbreviations: COPD, Chronic Obstructive Pulmonary Disease; FEV_1_, forced expiratory volume in 1 second; VC, vital capacity; *TGF-β*_*1*_, transforming growth factor-β1

* 0 means wild-type; 1 means minor allele

^# ^P value of Chi-square test for difference in prevalence of haplotype between subjects with and without COPD

### Lung function

We found no significant associations (i.e. cross-sectional) between the SNPs tested and FEV_1 _and FEV_1_/VC at the first or at the last survey in linear regression models (data not shown). The mean adjusted annual decline in lung function (expressed as decrease in FEV_1 _in ml/yr) was determined for subjects with the wild-type genotype for the SNPs in *TGF-β*_*1 *_and *decorin *using LME models. The outcome of the mean annual decline concerns females with age 30 when entered in the LME, a mean first FEV_1 _of the population, and zero pack-years. The mean of these adjusted annual declines was 19.2 ml/yr (range 18.7–19.6). We did not find any significant association of SNPs in either *TGF-β*_*1 *_or *decorin *with accelerated lung function decline (table 4). We added interaction terms of *TGF-β*_*1 *_en *decorin *SNPs in the model, but found no significant interactions. In addition, we did not find any significant association of haplotypes of either *TGF-β*_*1 *_or *decorin *with accelerated lung function decline (results not shown). We also tested whether SNPs were associated with lung function decline within subjects with COPD or within smokers, but found no significant associations (table 4 and additional file 4). To test whether results were not missed due to chance, we performed permutation tests. We ran 3000 permutations on our sample of 1390 subjects and performed LME analyses on each of these permutations. The lack of associations with lung function decline was confirmed in these analyses.

**Table 4:** Annual decline in FEV_1 _according to genotypes of *TGF-β*_*1 *_and *decorin*. Changes in decline between genotypes in the total population and in subjects who developed COPD (GOLD stage II or higher; FEV_1_/VC<70%, FEV_1_<80% predicted) are presented.

			**Total population**				**COPD**			
**Genotype**			**N**	Decline in FEV_1_(ml/yr)*	**ΔFEV_1 _compared to WT**	**P value†**	**N**	Decline in FEV_1_(ml/yr)*	**ΔFEV_1 _compared to WT**	**P value†**

**TGF-β_1_**	rs6957	AA	918	-19.2			103	-37.1		
		AG	399	-18.3	+0.9	0.511	71	-33.5	+3.6	0.297
		GG	40	-18.2	+1.0	0.778	10	-28.8	+8.3	0.239
										
	rs1800469	GG	716	-18.9			106	-34.3		
		GA	555	-17.6	+1.2	0.501	67	-36.2	-1.9	0.587
		AA	103	-20.3	-1.5	0.437	10	-31.9	+2.4	0.698
										
	rs1982073	GG	477	-19.1			75	-34.8		
		GA	623	-17.9	+1.2	0.309	72	+0.9	0.876	
		AA	185	-17.9	+1.2	0.593	23	-35.1	-0.3	0.959

**Decorin**	rs1803343	GG	1293	-18.7			173	-35.9		
		GA	85	-18.3	+0.4	0.874	13	-33.6	+2.3	0.698
										
	rs11106030	CC	1206	-18.9			170	-35.2		
		CA	162	-19.6	-0.7	0.688	8	-38.3	-3.1	0.577
		AA	6	-30.5	-11.6	0.285	1	-39.9	-4.7	0.797
										
	rs741212	AA	1039	-18.6			131	-35.1		
		AG	198	-20.1	-1.5	0.287	43	-38.2	-3.1	0.439
		GG	20	-14.1	+4.5	0.346	4	-23.2	+11.9	0.282
										
	rs516115	AA	737	-18.8			102	-34.4		
		AG	519	-18.5	+0.3	0.814	65	-35.9	-1.5	0.669
		GG	96	-18.9	+0.1	0.969	15	-35.0	-0.6	0.930
										
	rs3138241	GG	1187	-18.8			136	-35.7		
		GA	157	-19.5	-0.7	0.694	10	-38.7	-3.0	0.588
		AA	5	-25.7	-6.8	0.589	1	-31.6	+4.1	0.888

Abbreviations: FEV_1_, forced expiratory volume in 1 second; *TGF-β*_*1*_, transforming growth factor-β_1_; COPD, Chronic Obstructive Pulmonary Disease; WT, wild-type

*decline in FEV_1 _adjusted for gender, first FEV_1 _after age 30 years, pack-years, and age; † P value indicates significance of the effect of the genotype on decline in FEV_1 _compared to wild-type

## Discussion

Decorin and *TGF-β*_*1 *_can act as each other's feed back regulators in ECM turnover and their expression is respectively decreased and increased in lung tissue of COPD patients. We assessed whether polymorphisms in *decorin *and *TGF-β*_*1 *_are associated with the development of COPD and accelerated lung function decline in the general population. This is the first study assessing SNPs in *decorin *and we did not find any association with COPD or lung function loss. Contrary to our hypothesis, the observed disturbed balance between decorin and TGB-β_1 _in COPD is not caused by a combination of SNPs in their genes, since we found no significant interaction terms of *decorin *and *TGF-β*_*1 *_SNPs with respect to FEV_1 _decline. Moreover, we found no associations of phased multi-locus genotypes containing SNPs of both *TGF-β*_*1 *_and *decorin *with the presence of GOLD stage II and III COPD in our population. This disturbed balance may be affected by SNPs in *TGF-β*_*1 *_alone since the 3'UTR SNP in *TGF-β*_*1 *_is predictive of COPD (stage GOLD II). We found, however, no association of SNPs in *TGF-β*_*1 *_with longitudinal decline in lung function. In addition, no associations were observed of SNPs in *TGF-β*_*1 *_with level of FEV_1 _or FEV_1_/VC cross-sectionally.

It is puzzling that we observed that the *TGF-β*_*1 *_rs6957 SNP and a haplotype in *TGF-β*_*1 *_were associated with COPD, but not with excess decline in FEV_1 _or with level of FEV_1 _and FEV_1_/VC at the last survey. We have tested whether there were differences in first available FEV_1 _(which might suggest a relation to maximal attained lung function level) between the genotypes that could explain the lack of association with FEV_1 _decline but this was not the case. Another possibility would be that the FEV_1 _decline is only affected by SNPs in certain subgroups, such as smokers. Our stratified analyses showed no such effect.

Although the functionality of the *TGF-β*_*1 *_rs6957 SNP is not known yet, it has previously been associated with lower pre- and post-bronchodilator FEV_1 _and with lower FEV_1_/FVC.[14] Similarly, we have shown here that this SNP is associated with development of COPD. Various studies have indicated that the rs1800469 and rs1982073 SNPs are functional and result in higher levels of circulating TGF-β_1_. [23-26] Since TGF-β_1 _has anti-inflammatory and pro-repair activities, these SNPs are thought to be protective against the development of COPD. Indeed, we and others have found that (carriers of haplotypes of) the minor alleles of these SNPs are significantly less prevalent in COPD patients compared to controls.[14,16]. Similar to Celedón *et al*, we found an association of a haplotype with at least one minor allele of the rs1800469 and rs1982073 *TGF-β*_*1 *_SNPs and COPD, while they also found associations with these SNPs separately. [14,16] The differences in study populations may explain these dissimilarities, e.g. our subjects had milder COPD (FEV_1_<80% predicted) than the COPD patients in the Celedón study (FEV_1_<45% predicted). Despite the differences in associations, it is still conceivable that carrying both of the SNPs decreases the risk to develop COPD. The two other studies linking *TGF-β*_*1 *_SNPs and COPD have also demonstrated that these SNPs are less prevalent in COPD, though these studies did not test haplotypes[15,16]

Many SNPs have been described in the *TGF-β*_*1 *_gene, but only a few have been intensively studied in genetic association studies. Cross-sectional studies have found associations of SNPs in *TGF-β*_*1 *_with the presence of COPD, and with lower levels of FEV_1 _and FEV_1_/FVC in several populations. [14-16] We did not analyze every SNP in the *TGF-β*_*1 *_gene that was previously reported to be associated with COPD. However, since Celedón *et al *found strong LD (r^2 ^= 0.98) between promoter SNPs and 3'UTR SNPs in a Caucasian population, we are confident that any association that might exist would have been revealed by the SNPs or by their haplotypes.[14]

This is the first study on SNPs in *decorin *in a general population or in COPD patients. We were interested in polymorphisms in this gene, since decorin expression in COPD patients is diminished.[9,10] Decorin plays a direct role in the repair processes after inflammation through its regulation of matrix metalloproteases and tissue inhibitors of metalloproteases.[27,28] Furthermore, decorin is the natural inhibitor of *TGF-β*_*1 *_and may therefore influence the repair process in the lung indirectly. We hypothesized that these processes may be genetically influenced. Since the coding SNPs in decorin described in the NCBI and Celera databases were not prevalent in Caucasians (but only in African populations), we genotyped four tagging SNPs, located in introns, and additionally a 3'UTR SNP. Although we found no significant associations of these SNPs with COPD or lung function decline, we can not rule out completely that there is no genetic defect in *decorin *that increases the risk to develop COPD. However, since we selected tagging SNPs that cover the genetic information of the decorin gene according to HapMap and given the large population under study, we assume that we would have observed an association of SNPs or haplotypes in *decorin *if there existed one in this population.

The lack of a genetic association of SNPs in the *decorin *gene does not rule out an important role of the decorin protein in COPD development. Decorin is a member of the proteoglycan family, a family of macromolecules composed of a protein core with glycosaminoglycan side chains which are produced post-translationally. It is possible that the function or activation of decorin is disrupted through an altered posttranslational modification of this glycosaminoglycan chain. In this case, modifications in the protein core, which might be caused by SNPs, may not be important and will not be detected. Decorin can be expressed in six splice variants, but the function of these splice variants is not known yet. Nevertheless, a shift in prevalence of one of these splice variants may affect the biological role that decorin exerts in *TGF-β*_*1 *_regulation, thereby influencing the pathology within the lung.

## Conclusion

Contrary to our hypothesis, we were not able to identify the *decorin *gene as a genetic risk factor for the development of COPD. Consequently, SNPs in *decorin *do not seem to underlie a disturbed regulation of this gene and *TGF-β*_*1 *_resulting in COPD, nor can they be held responsible for the development of COPD and decline in FEV_1_in the general population. We found that *TGF-β*_*1 *_SNPs are associated with the development of COPD but not with accelerated lung function decline or other lung function measures in the general population. Together with previous findings, this study establishes the *TGF-β*_*1 *_gene as a risk factor for the development of COPD.

## Competing interest statement

The author(s) declare that they have no competing interests.

## Authors' contributions

Every author contributed to reviewing of the paper. CCD performed the lab work, statistical analyses and drafted the manuscript. DSP is co principal investigator of the project, obtained funding of and supervised the project, and helped draft the manuscript. JMV contributed to the statistical analyses. MB contributed to the lab work. IMN contributed to the statistical analyses. HMB is co principal investigator of the project, obtained funding of and supervised the project, and helped draft the manuscript. All authors read and approved the final manuscript.

## Supplementary Material

Additional File 1 **Methods**. Detailed description of the pulmonary function protocol and the genotyping protocol Click here for file

Additional File 2 **Characteristics of genotyped SNPs**. Table with specifications of the genotyped SNPs, i.e. location, characteristics and sequences of primers and probes. Click here for file

Additional File 3 Linkage Disequilibrium of SNPs in *decorin *and *TGF-β*_*1*_. Click here for file

Additional File 4 **Annual decline in FEV_1 _according to genotypes of *TGF-β*_*1 *_and *decorin***. Changes in decline between genotypes in never smokers and current and past smokers are presented. Click here for file
