# Supplementary material for: Decorin and TGF-β1 polymorphisms and development of COPD in a general population
Source: Respir Res. 2006 Jun 16;7(1):89. doi: 10.1186/1465-9921-7-89 (PMC1539000; doi:10.1186/1465-9921-7-89)
Supplement: Additional File 1 — Methods. Detailed description of the pulmonary function protocol and the genotyping protocol [file 1465-9921-7-89-S1.doc]

### Additional file 1 – Methods

**Pulmonary function measurements**

Pulmonary function measurements were performed with a water sealed spirometer (Lode Spirograph D53, Lode Instruments, Groningen, The Netherlands). An inspiratory vital capacity (IVC) was measured after a deep expiration and followed by measurement of forced expiratory volume in 1 second (FEV_1_). Subjects performed the manoeuvre until two technically satisfactory tracings were produced. The higher value of the two tracings was taken as the baseline measurement. For a tracing to be acceptable, the difference between two IVC values could not be more than 150 ml, and the difference between two FEV1 measurements not more than 100 ml. All values were recorded at ATPS. The surveys always took place in October.

**Genotyping protocol**

Primers and probes of all SNPs were obtained from Applied Biosystems TaqMan® SNP Genotyping Assays (Nieuwekerk aan de IJssel, The Netherlands), using the Assay-by-Design service, for which we provided sequences, or the Assay-on-demand service when assays were already designed by Applied Biosystems. Reactions were performed in 5 μl volumes and contained 10 ng DNA, 1x Taqman Universal Mastermix (Applied Biosystems), 200 nM of each probe and 900 nM of each primer. Cycling conditions on the ABI PRISM® 7900HT Sequence Detection System (Applied Biosystems) were 10 min 95ºC followed by 40 cycles of 15 seconds 95ºC and 1 minute 60ºC. End-point fluorescence was measured after cycling. Alleles were assigned using SDS 2.1 software (Applied Biosystems). Genotyping reactions failed randomly with a minimum of 0.9% and a maximum percentage of 7.6% per SNP. There was no relationship between degree of missing genotypes and evidence of association. We regenotyped 6% of the samples and found no errors in any of the genotypes.
