# Supplementary material for: Decorin and TGF-β1 polymorphisms and development of COPD in a general population
Source: Respir Res. 2006 Jun 16;7(1):89. doi: 10.1186/1465-9921-7-89 (PMC1539000; doi:10.1186/1465-9921-7-89)
Supplement: Additional File 2 — Characteristics of genotyped SNPs. Table with specifications of the genotyped SNPs, i.e. location, characteristics and sequences of primers and probes. [file 1465-9921-7-89-S2.doc]

Additional file 2- Characteristics of genotyped SNPs

| **Gene** | **rs number** | **Location of SNP in gene** | **MAF** | **Functionality** | **AB assay** | **Primers*** | **Probes*** |
| --- | --- | --- | --- | --- | --- | --- | --- |
| TGF-β1 | rs1800469 | promoter -509 | 0.282 | Ass. with increased TGF-β_1_ levels | - | For: GGAGAGCAATTCTTACAGGTGTCT  Rev: GGAGAAGAGGGTCTGTCAACATG | Vic: ACACCTGAGGGATGG  Fam: ACACCTGAAGGATGG |
| TGF-β1 | rs1982073 | exon 1, +29 | 0.376 | Ass. with increased levels of TGF-β_1_ in serum | - | For: CGCGCTCTCGGCAGT  Rev: AGGCGTCAGCACCAGTAG | Vic: CAGCAGCGGCAGCA  Fam: CAGCAGCAGCAGCA |
| TGF-β1 | rs6957 | locus 28344 3’UTR | 0.178 | unknown | C_7818385_10 |  |  |
| Decorin | rs11106030 | 5’UTR | 0.08 | unknown | - | For: ACATTTAATGTGGGCTGTCAATACTGA  Rev: GGACAGCTGCTCTTTGCTCTTTATA | Vic: GACAGCTGCTCTTTGCTCTTTATA  Fam: CTTTCGTCATTTCC |
| Dec­­orin | rs741212 | 5’UTR | 0.120 | unknown | C_2309576_10 |  |  |
| Decorin | rs566806 | intron 1 | 0.262 | unknown | C_2675824_10 |  |  |
| Decorin | rs3138241 | intron 2 | 0.06 | unknown | C_30790690 |  |  |
| Decorin | rs1803343 | 3’UTR | 0.024 | unknown | C_7561184_10 |  |  |

Abbreviations: SNP, Single Nucleotide Polymorphism; MAF, minor allele frequency; AB assay, Applied Biosystems assay on demand; TGF-β_1_, transforming growth factor-β_1_; UTR, untranslated region;

Sequences of primers and probes are listed when the assay was designed by AB, otherwise sequences are known by AB
