# Supplementary figures and images for: Decorin and TGF-β1 polymorphisms and development of COPD in a general population
Source: Respir Res. 2006 Jun 16;7(1):89. doi: 10.1186/1465-9921-7-89 (PMC1539000; doi:10.1186/1465-9921-7-89)

**Additional file 3: Linkage Disequilibrium of SNPs in *decorin* and *TGF-β1***

***Decorin:***


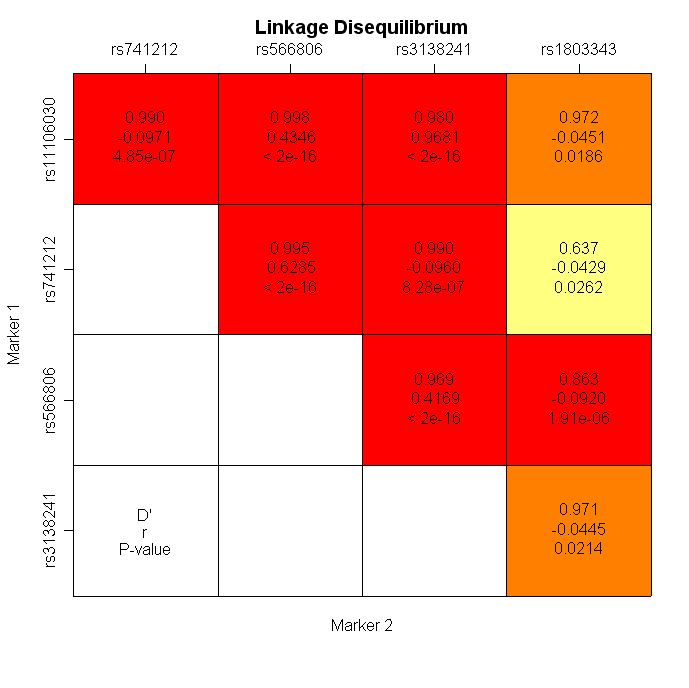


***TGF-β1:***


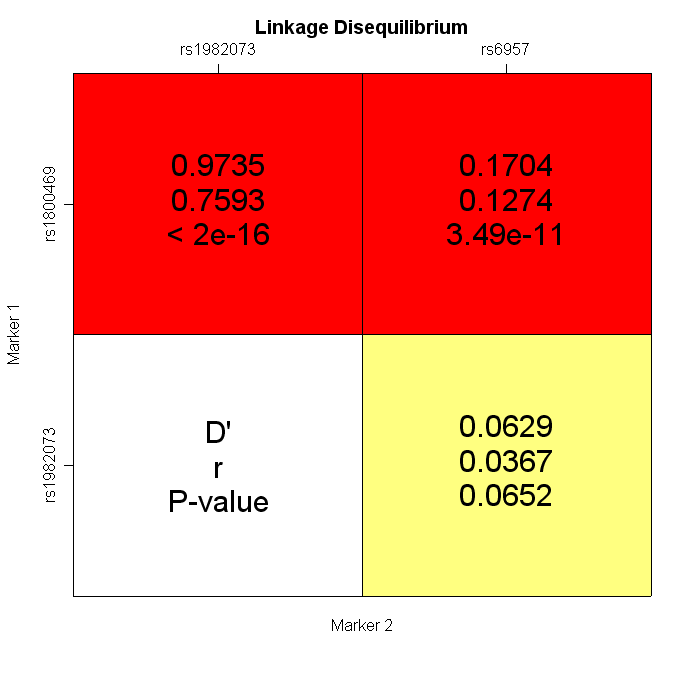

Supplement: Additional File 3 — Linkage Disequilibrium of SNPs in decorin and TGF-β1. [file 1465-9921-7-89-S3.doc]
