# Supplementary material for: Decorin and TGF-β1 polymorphisms and development of COPD in a general population
Source: Respir Res. 2006 Jun 16;7(1):89. doi: 10.1186/1465-9921-7-89 (PMC1539000; doi:10.1186/1465-9921-7-89)
Supplement: Additional File 4 — Annual decline in FEV1 according to genotypes of TGF-β1 and decorin. Changes in decline between genotypes in never smokers and current and past smokers are presented. [file 1465-9921-7-89-S4.doc]

**Additional file 4: Association of annual decline in FEV_1_ according to genotypes of *TGF-β_1_* and *decorin***. Changes in decline between genotypes in never smokers and current and past smokers.

|  | | | **Decline in FEV_1_ (ml/yr)^*^** | |  | **Decline in FEV_1_ (ml/yr)^*^** | |  |
| --- | --- | --- | --- | --- | --- | --- | --- | --- |
| **Genotype** | | | **Never smokers** | **ΔFEV_1_ compared to WT** | **P value**† | **Ever smokers** | **ΔFEV_1_ compared to WT** | **P value**† |
| **TGF-β_1_** | rs6957 | AA | -20.4 |  |  | -21.7 |  |  |
|  |  | AG | -21.0 | -0.6 | 0.736 | -19.5 | +2.2 | 0.230 |
|  |  | GG | -25.3 | -4.9 | 0.312 | -18.1 | +3.6 | 0.426 |
|  |  |  |  |  |  |  |  |  |
|  | rs1800469 | GG | -21.3 |  |  | -20.8 |  |  |
|  |  | GA | -20.2 | +1.1 | 0.512 | -19.6 | +1.2 | 0.498 |
|  |  | AA | -20.6 | +0.7 | 0.831 | -19.6 | +1.2 | 0.712 |
|  |  |  |  |  |  |  |  |  |
|  | rs1982073 | GG | -21.3 |  |  | -20.2 |  |  |
|  |  | GA | -20.0 | +1.3 | 0.588 | -19.2 | +1.0 | 0.673 |
|  |  | AA | -20.9 | +0.4 | 0.864 | -22.4 | -2.2 | 0.402 |
|  |  |  |  |  |  |  |  |  |
| **Decorin** | rs1803343 | GG | -21.0 |  |  | -30.3 |  |  |
|  |  | GA | -23.2 | -2.2 | 0.469 | -17.7 | +2.6 | 0.421 |
|  |  |  |  |  |  |  |  |  |
|  | rs11106030 | CC | -20.9 |  |  | -20.4 |  |  |
|  |  | CA | -21.2 | -0.3 | 0.918 | -21.1 | -0.7 | 0.793 |
|  |  | AA | -29.5 | -8.6 | 0.594 | -29.8 | -9.3 | 0.511 |
|  |  |  |  |  |  |  |  |  |
|  | rs741212 | AA | -21.2 |  |  | -19.9 |  |  |
|  |  | AG | -20.5 | +0.7 | 0.708 | -22.7 | -2.8 | 0.165 |
|  |  | GG | -18.3 | +2.9 | 0.533 | -9.5 | +10.4 | 0.246 |
|  |  |  |  |  |  |  |  |  |
|  | rs566806 | AA | -21.3 |  |  | -20.9 |  |  |
|  |  | AG | -21.3 | -0.0 | 0.992 | -20.1 | +0.8 | 0.639 |
|  |  | GG | -20.2 | +1.1 | 0.691 | -19.6 | +1.3 | 0.701 |
|  |  |  |  |  |  |  |  |  |
|  | rs516115 | AA | -21.4 |  |  | -20.5 |  |  |
|  |  | AG | -21.1 | +0.3 | 0.873 | -19.6 | +0.9 | 0.613 |
|  |  | GG | -20.6 | +0.8 | 0.779 | -20.7 | -0.2 | 0.849 |
|  |  |  |  |  |  |  |  |  |
|  | rs3138241 | GG | -20.9 |  |  | -20.3 |  |  |
|  |  | GA | -21.2 | -0.3 | 0.881 | -20.8 | -0.5 | 0.865 |
|  |  | AA | -29.3 | -8.4 | 0.598 | -21.0 | -0.7 | 0.969 |
|  |  |  |  |  |  |  |  |  |

Abbreviations: FEV_1_, forced expiratory volume in 1 second; TGF-β_1_, transforming growth factor-β_1_; COPD, Chronic Obstructive Pulmonary Disease; WT, wild-type

^*^decline in FEV_1_ adjusted for gender, first FEV_1_ after age 30 years, and age; † P value indicates significance of the effect of the genotype on decline in FEV_1_ compared to wild-type
